# Single-Stranded DNA-Binding Proteins Mediate DSB Repair and Effectively Improve CRISPR/Cas9 Genome Editing in *Escherichia coli* and *Pseudomonas*

**DOI:** 10.3390/microorganisms11040850

**Published:** 2023-03-27

**Authors:** Ran Chai, Qi Zhang, Jie Wu, Ziwen Shi, Yanan Li, Yuqian Gao, Yuancheng Qi, Liyou Qiu

**Affiliations:** College of Life Sciences, Henan Agricultural University, Key Laboratory of Enzyme Engineering of Agricultural Microbiology, Ministry of Agriculture and Rural Affairs, Zhengzhou 450046, China

**Keywords:** single-stranded DNA-binding protein, DNA double-strand breaks, dsDNA repair, CRISPR/Cas9 genome editing, homologous recombination, nonhomologous end joining

## Abstract

Single-stranded DNA-binding proteins (SSBs) are essential for all living organisms. Whether SSBs can repair DNA double-strand breaks (DSBs) and improve the efficiency of CRISPR/Cas9-mediated genome editing has not been determined. Here, based on a pCas/pTargetF system, we constructed pCas-SSB and pCas-T4L by replacing the λ-Red recombinases with *Escherichia coli* SSB and phage T4 DNA ligase in pCas, respectively. Inactivation of the *E. coli lacZ* gene with homologous donor dsDNA increased the gene editing efficiency of pCas-SSB/pTargetF by 21.4% compared to pCas/pTargetF. Inactivation of the *E. coli lacZ* gene via NHEJ increased the gene editing efficiency of pCas-SSB/pTargetF by 33.2% compared to pCas-T4L/pTargetF. Furthermore, the gene-editing efficiency of pCas-SSB/pTargetF in *E. coli* (Δ*recA*, Δ*recBCD*, Δ*SSB*) with or without donor dsDNA did not differ. Additionally, pCas-SSB/pTargetF with donor dsDNA successfully deleted the *wp116* gene in *Pseudomonas* sp. UW4. These results demonstrate that *E. coli* SSB repairs DSBs caused by CRISPR/Cas9 and effectively improves CRISPR/Cas9 genome editing in *E. coli* and *Pseudomonas*.

## 1. Introduction

CRISPR/Cas gene-editing technology has been rapidly adopted and widely used in microorganisms, plants, animals, and humans since it was established in 2012 [1]. CRISPR is an acronym for clustered regularly interspaced short palindromic repeats, which comprise a leader sequence, multiple repeats, and spacers [2,3]; these repeats are present in the genomes of 42.3% of bacteria and 85.2% of archaea [4]. CRISPR-adjacent regions bear CRISPR-associated (Cas) genes that encode a series of Cas nucleases. When a phage or exogenous gene invades a bacterium, the Cas protein can recognize the protospacer adjacent motif (PAM) sequence, excise its upstream protospacer, and insert it between the leader sequence and the adjacent repeat to form a new spacer [5]. The foreign homologous sequence subsequently invades, and the promoter in the leader sequence initiates transcription of the CRISPR array, producing a long-stranded RNA called the precursor transcript (pre-crRNA). The pre-crRNA is then processed by Cas proteins into mature crRNA, which contains a single spacer. crRNA guides the Cas protein, which cuts homologous DNA and eliminates the invading foreign DNA [6]. CRISPR/Cas gene-editing technology is derived from this adaptive immune defense mechanism that occurs in bacteria.

A small-molecular-weight Cas protein (SpCas9) from *Streptococcus pyogenes* can complete DNA splicing alone, requiring a relatively common PAM sequence [7]. However, to splice its target DNA, SpCas9 requires the guidance of guide RNA (gRNA) or single-translated guide RNA (sgRNA), which is formed by the complementary pairing of crRNA and the noncoding trans-activating CRISPR RNA (tracrRNA) gene transcript. The tracrRNA gene is also located in the CRISPR-adjacent region [8]. The reconstitution of SpCas9 and an artificial chimera of crRNA and tracrRNA, which is called synthetic-guide RNA (sgRNA), greatly simplifies the process of gene editing, thus increasing the application of the Cas9/sgRNA system.

In recipient cells, Cas9/sgRNA sufficiently cuts target genes to produce DNA double-strand breaks (DSBs); subsequently, the cells repair these DSBs by nonhomologous end joining (NHEJ) or homologous recombination (HR) repair, generating point mutations. Eukaryotes use canonical DNA-PKcs-dependent NHEJ (D-NHEJ) [9,10], alternative NHEJ (alt-NHEJ) [11], or RAD51/BRCA2-dependent HR to repair DSBs [12]. HR repair can only occur during the S/G2 phase and requires homologous donor DNA, while NHEJ repair is constitutively active throughout the entire cell cycle, does not require donor DNA, and generates nucleotide insertions or deletions at the cleavage site. Therefore, NHEJ repair is the dominant pathway that is used for DSB repair in eukaryotic cells [13,14].

To repair DSBs during the logarithmic phase of growth, prokaryotes use RecA-dependent HR repair [15]; during the stationary phase of growth, several bacteria, excluding *E. coli* [16], use Ku- and Ligase D-dependent NHEJ repair [17,18], while *E. coli* uses RecBCD and LigA-dependent NHEJ, which is called alternative end-joining (A-EJ) [19]. However, the use of the CRISPR/Cas9 system in bacteria faces challenges. The use of Cas9- and gRNA-expressing plasmids or the addition of homologous DNA, even in the absence of crRNA or inactive Cas9 (dCas9), results in few or no surviving colonies or in abnormal morphology and decreased growth rates [20,21,22,23,24]. It has been suggested that DSBs, transient PAM recognition, and enzymatic binding along the DNA have cytotoxic effects on prokaryotic cells [22,25], and DSB repair by native HR and NHEJ repair mechanisms exhibit very low efficiency in bacteria.

The use of inducible Cas9 expression systems [26,27] or the incorporation of λ-Red homologous recombination enzymes and homologous recombination templates [26,27], the mycobacteria-derived Ku- and Ligase D-dependent NHEJ pathway [28], *E. coli*-derived RecBCD + LigA [29], the λ phage-derived Redβ protein [30], the *Pseudomonas putida*-derived Ssr protein [31] and the T4 DNA ligase [32] produce surviving colonies and result in efficient editing. Jiang et al. developed a facile bacterial gene-editing system, namely, the pCas/pTargetF system [33]. In this system, the pCas plasmid harbors the λ-Red recombination system, and the IPTG-inducible expression of sgRNA-PMB1 facilitates the guiding of the pMB1-dependent replication of pTarget to eliminate pTargetF. pCas is later eliminated by incubation at 37 °C because it harbors a temperature-sensitive replicon. pTargetF carries the sgRNA expression cassette. The λ-Red recombination system consists of the Exo (Redα), Beta (Redβ), and Gam (Redγ) proteins. Exo is a 5’ to 3’ dsDNA-dependent exonuclease that generates a ssDNA intermediate. Beta is a ssDNA-binding protein that promotes pairing or annealing between complementary ssDNAs. Gam prevents the degradation of dsDNA to enhance recombination by inhibiting the cellular nucleases [34]. Employing the pCas/pTargetF system to edit the single gene locus in *E. coli*, the efficiency of gene editing is 6–92%, which very much depends on the homologous arm length of donor DNA and donor DNA supplied in pTarget or in PCR fragment [33]. The pCas/pTargetF system has been used in a variety of bacteria [35,36,37,38].

Single-stranded DNA-binding proteins (SSBs) are essential for all living organisms and viruses [39]. SSBs bind to single-stranded DNA (ssDNA) with high affinity and in a sequence-independent manner in order to prevent the transient formation of dsDNA from ssDNA and to protect ssDNA from undesirable damage by nucleases and chemicals [40,41]. Furthermore, SSBs interact with every protein that is involved in DNA metabolism and thus participate in DNA replication, recombination, and repair as well as genome maintenance [42,43]. Previously, an SSB in *E. coli* has been shown to possess the ability to catalyze both DNA HR and nonhomologous recombination (NHR) [44]. Whether this protein can promote bacterial DSB repair during CRISPR/Cas9-mediated gene editing and improve gene-editing efficiency has not been determined. In this study, SSB was used to replace the λ-Red recombination system in pCas and was confirmed to efficiently promote HR- and NHR-mediated gene editing by the pCas/pTargetF system in *E. coli* and *Pseudomonas*.

## 2. Material and Methods

### 2.1. Strains, Plasmids and Culture Conditions

The bacterial strains and plasmids that were used in this study are listed in Table 1. The strains were grown in Luria–Bertani broth (LB) medium (1% (*w*/*v*) tryptone, 0.5% (*w*/*v*) yeast extract, 1% (*w*/*v*) NaCl) at 37 °C or 30 °C. Kanamycin (25 μg/mL), spectinomycin (50 μg/mL), or tetracycline (15 μg/mL) were added as needed. *E. coli* DH5α was used for plasmid construction and maintenance, and *E. coli* MG1655 and *Pseudomonas* sp. UW4 were used for gene editing procedures.

### 2.2. Plasmid Construction

All the primers that were used in this study are listed in Table 2. To construct the pCasΔ*Red* plasmid, the pCas plasmid was amplified by PCR using the pC01/pC02 primers to generate a linear DNA fragment without the λ-Red recombination system. The resulting linear DNA fragment was self-ligated to form a circular plasmid by using the ClonExpress^®^ II One Step Cloning Kit (Vazyme, Nanjing, China), resulting in the generation of pCasΔ*Red*. To construct the pCas-SSB plasmid, the SSB gene, which contained the sequences that are homologous to the upstream and downstream regions of the λ-Red recombination system in pCas, was amplified by PCR using *E. coli* DH5α genomic DNA as a template and the pC03/pC04 primers. The linear fragment of pCas was amplified by PCR using pCas as a template and the pC05/pC06 primers. The two resulting DNA fragments were processed by using a ClonExpress^®^ II One Step Cloning Kit to generate pCas-SSB. The SSB gene sequence was recorded in GenBank (accession no. 948570). Similarly, the pCas-T4L plasmid was constructed by replacing the SSB gene with the T4 DNA ligase gene with the pC07/pC08 primers. The T4 DNA ligase gene-template DNA was synthesized by BGI (Shenzhen, China) according to the sequence in GenBank (accession no. 1258680).

pTargetF-*lacZ* and pTargetF-*wp116* were constructed by primer site mutation of pTargetF using the Fast Mutagenesis System kit (TransGen Biotech, Beijing, China), following the manufacturer’s protocol. The whole pTargetF plasmid was amplified by using the pC09/pC10 and pC11/pC12 primers. After digestion with the DMT enzyme, the PCR products were transformed into *E. coli* DH5α. The plasmids were extracted, and sequencing confirmed that they were pTargetF-*lacZ* and pTargetF-*wp116*.

### 2.3. Donor DNA Construction

Donor DNAs were used to delete the *lacZ* gene in *E. coli* MG1655 and the *wp116* gene in *Pseudomonas* sp. UW4 via HR-mediated genome editing. The donor DNAs had 200-bp and 140-bp sequences that were homologous to each side (upstream or downstream) of the target region in the genome. The donor DNA that was used to delete the *lacZ* gene was constructed by overlapping PCR of the two fragments that were amplified by using *E. coli* MG1655 genomic DNA as a template and the pC13/pC14 and pC15/pC16 primers. The donor DNA, which harbored a tetracycline-resistance gene cassette that was used to delete the *wp116* gene, was constructed by overlapping PCR of the three fragments that were amplified using *Pseudomonas* sp. UW4 Δ*acdS* genomic DNA as a template and the pC17/pC18, pC19/pC20, and pC21/pC22 primers. The *wp116* gene encodes a putative methyl-accepting chemotaxis protein of UW4 (GenBank accession no. WP_015093116).

### 2.4. Genome Editing

pCas/pTargetF, plasmids derived from pCas/pTargetF and donor DNA as needed, were co-transformed into *E. coli* strains according to a standard CaCl_2_ transformation procedure [48] and into *Pseudomonas* sp. UW4 by electroporation as previously described [49]; both procedures were conducted with slight modification. Two hundred nanograms of each plasmid and 400 ng of donor DNA as needed were transfected into a 100 μL suspension of competent cells. After heat shock or electroporation, the mixture was immediately added to 0.9 mL of fresh LB medium supplemented with 10 mM arabinose and, then, the cells were allowed to recover by incubation at 30 °C and 220 rpm for 2 h. Then, the culture was divided into three aliquot parts and spread on three LB plates supplemented with 1 mM IPTG, 40 μg/mL X-gal, and 25 μg/mL kanamycin, 50 μg/mL spectinomycin, or 15 μg/mL tetracycline as needed. The plates were incubated overnight at 37 °C. The transformation experiment was conducted thrice for replication.

Transformants were identified by blue and white plaque assay, colony PCR, and DNA sequencing. The white colonies of *E. coli lacZ* mutation transformants were identified by PCR using the pC23/pC24 primer pair for HR and the pC25/pC26 primer pair for HNEJ. The resulting PCR products were also subjected to sequencing using the primer pairs. The pC27/pC28 primer pair was used to identify the *wp116* HR transformants of *Pseudomonas* sp. UW4 by PCR and sequencing the resulting PCR products.

The two CRISPR plasmids that were used in this study endowed their host bacteria with kanamycin and spectinomycin resistance. Thus, the transformation rate was calculated by dividing the number of colonies that survived on LB kanamycin and spectinomycin plates by the quantity of one plasmid DNA. The efficiency of *E. coli lacZ* gene mutation by genome editing via CRISPR/Cas9-mediated NHEJ indel or HR integration was determined by calculating the ratio of white colonies to total colonies (white + blue colonies) on LB plates that were supplemented with 40 μg/mL X-gal and 25 μg/mL kanamycin or 50 μg/mL spectinomycin. The efficiency of the *Pseudomonas* sp. UW4 *wp116* gene mutation by genome editing is expressed as the percentage of colonies with an altered target gene sequence among 20 randomly selected colonies.

## 3. Results

### 3.1. SSB Mediated the HR Repair of DSBs and Improved the Efficiency of CRISPR/Cas9 in Deleting the E. coli lacZ Gene

**Figure 1:**
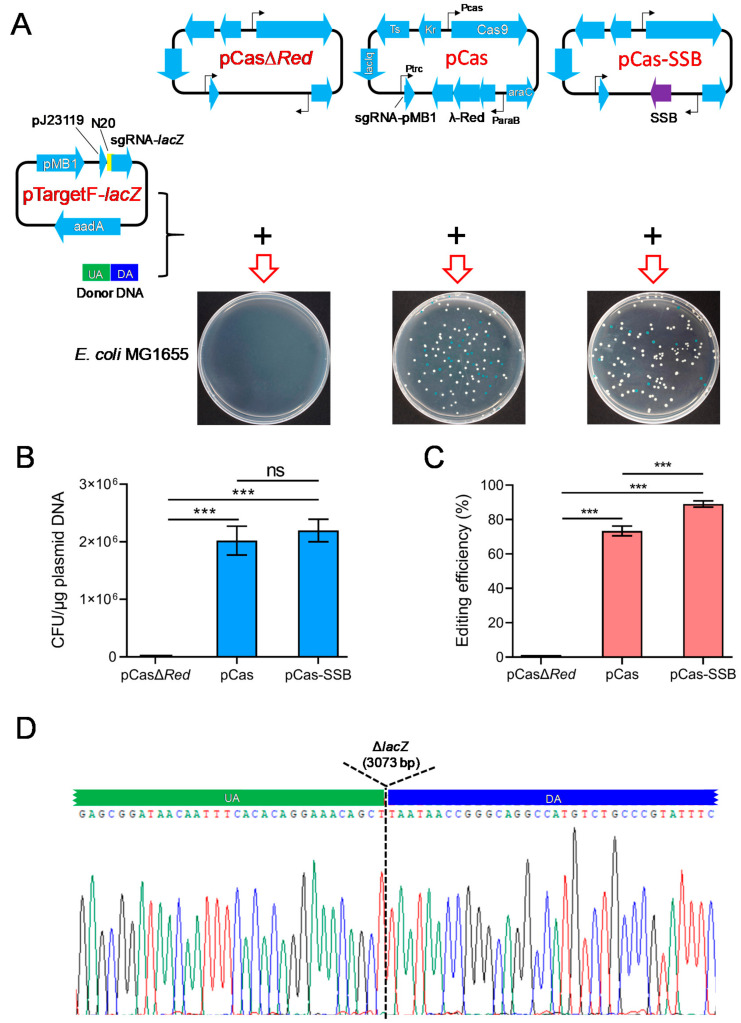
Comparison of CRISPR/Cas9 gene editing to delete the *E. coli lacZ* gene, assisted by SSB- and λ-Red recombination system-mediated HR repair of DSBs. (**A**) Schematic representation of the CRISPR-Cas9 two-plasmid system and the results of transforming the two plasmids into *E. coli* MG1655. LB plates were used containing X-gal, IPTG, kanamycin, and spectinomycin. pCas harbors *Streptococcus pyogenes* Cas9 under its native promoter Pcas, the λ-Red recombination system (λ-Red) with the arabinose-inducible promoter ParaB and arabinose-inducible transcription factor (araC), sgRNA-PMB1 with an IPTG-inducible promoter (Ptrc) guiding the pMB1 replication of pTarget for eliminating pTargetF, the *lac* repressor (lacIq), the temperature-sensitive origin of replication *repA101* (Ts), and kanamycin resistance gene (Kr); pCasΔ*Red* is derived from pCas by deletion of λ-Red; and pCas-SSB is derived from pCas by replacing λ-Red recombinases with *E. coli* SSB. pTargetF-*lacZ* contains sgRNA-*lacZ* with the efficient constitutive promoter pJ23119 targeting *lacZ* guided by N_20_ (Table 2), aadA conferring spectinomycin resistance and the pMB1 replicon; and the donor DNA contains an upstream homologous arm (UA) and a downstream homologous arm (DA) that are homologous to each side (upstream or downstream) of the *lacZ* gene in the *E. coli* genome. (**B**) The transformation rate of the CRISPR-Cas9 two-plasmid system into *E. coli* MG1665. (**C**) The editing efficiency of CRISPR-Cas9 to delete the *E. coli lacZ* gene. (**D**) Identification of the 10 randomly selected *lacZ* mutant colonies (white colonies) by colony PCR and DNA sequencing. The data in (**B**,**C**) represent the mean ± s.d. for *n* = 3 biologically independent samples. ***, *p* < 0.001 by one-way ANOVA followed by Dunnett’s test; ns, no significant differences.

To investigate whether SSB effectively mediates the HR repair of DSBs and improves the efficiency of CRISPR/Cas9 gene editing in *E. coli*, donor DNA carrying sequences that were homologous to the upstream and downstream regions of the *E. coli lacZ* gene as well as pCasΔ*Red*/pTargetF-*lacZ*, pCas/pTargetF-*lacZ*, or pCas-SSB/pTargetF-*lacZ* were transformed into *E. coli* MG1655. In the culture that was transformed with donor DNA and pCasΔ*Red*/pTargetF-*lacZ*, no colonies grew on the LB plates supplemented with X-gal, IPTG, kanamycin, and spectinomycin. In the culture that was transformed with donor DNA as well as pCas/pTargetF-*lacZ* or pCas-SSB/pTargetF-*lacZ*, white and blue colonies grew on the plates (Figure 1A). White colonies represented colonies in which the *lacZ* gene had been mutated, and blue colonies represented colonies without *lacZ* gene mutation. The transformation rates of the two cultures were greater than 2 × 10^6^ CFU/μg plasmid DNA, and there was no difference between the transformation rates of the two cultures. The gene editing efficiency of the two transformed cultures was 73.4% and 89.1%, and the gene editing efficiency observed in the culture that was transformed with pCas-SSB/pTargetF-lacZ and donor DNA was 21.4% higher than that in the culture that was transformed with pCas/pTargetF-lacZ and donor DNA (*p* < 0.001) (Figure 1B,C). In each transformation group, 10 white colonies were randomly picked from the plates and were subjected to colony PCR sequencing for identification. In all the mutant transformants, the *lacZ* gene was correctly knocked out by HR (Figure 1D).

### 3.2. Cas9/SSB Mediated NHEJ to Delete the E. coli lacZ Gene

**Figure 2:**
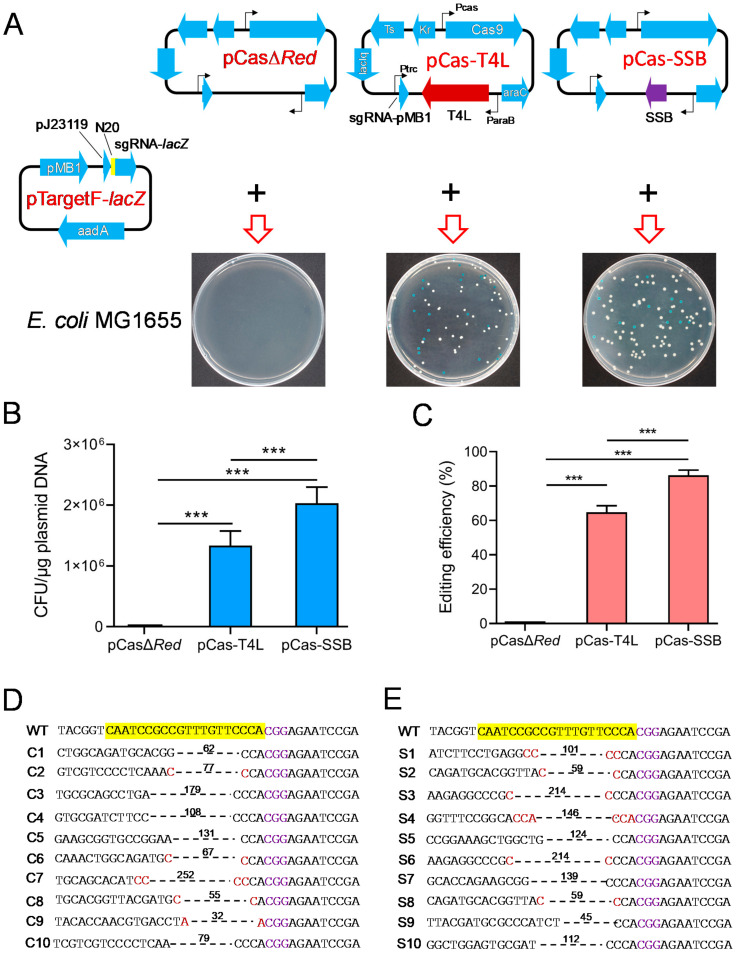
Effects of SSB- and T4 DNA ligase-mediated NHR repair of DSBs on CRISPR-Cas9 gene editing to delete the *E. coli lacZ* gene. (**A**) Illustration of Cas9 and sgRNA expression plasmids and their transformation into *E. coli* MG1655. pCas-T4L is derived from pCas by replacing λ-Red recombinases with phage T4 DNA ligase. (**B**) The transformation rate of the CRISPR-Cas9 plasmids into *E. coli* MG1665. (**C**) The editing efficiency of CRISPR-Cas9 to delete the *E. coli lacZ* gene. (**D**) Identification of the 10 randomly selected white colonies generated by pCas-T4L and pTargetF-*lacZ* transformation by colony PCR and sequencing. (**E**) Identification of the 10 randomly selected white colonies generated by pCas-SSB and pTargetF-*lacZ* transformation by colony PCR and sequencing. The sequences highlighted in yellow represent a 20-bp complementary region (N_20_) matching with the *E. coli lacZ* gene. The sequences colored in purple indicate the PAM sequence of Cas9. The sequences colored in red are micro-homology sequences. The dotted lines and associated numbers indicate the deleted sequences and deletion sizes (bp). WT, *E. coli* MG1665; C1-C10 or S1-S10, the 10 randomly selected white colonies. The data in (**B**,**C**) represent the mean ± s.d. for *n* = 3 biologically independent samples. *** *p* < 0.001 by one-way ANOVA followed by Dunnett’s test; ns, no significant differences.

Phage T4 DNA ligase can repair the chromosomal DNA DSBs that are generated by CRISPR/Cas9 through NHEJ [32]. To determine whether SSB effectively mediates NHEJ to promote CRISPR/Cas9 gene editing in *E. coli*, pCasΔ*Red*/pTargetF-*lacZ*, pCas-T4L/pTargetF-*lacZ*, and pCas-SSB/pTargetF-*lacZ* were transformed into *E. coli* MG1655. In the culture that was transformed with pCasΔ*Red*/pTargetF-*lacZ*, no colonies grew on the LB plates supplemented with X-gal, IPTG, kanamycin, and spectinomycin. In the culture that was transformed with pCas-T4L/pTargetF-*lacZ* or pCas-SSB/pTargetF-*lacZ*, white and blue colonies grew on the plates (Figure 2A). The transformation rates of the two cultures were 1.3 × 10^6^ CFU/μg plasmid DNA and 2.0 × 10^6^ CFU/μg plasmid DNA. The pCas-SSB/pTargetF-*lacZ* transformation rate was 52.6% higher than the pCas-T4L/pTargetF-*lacZ* transformation rate (*p* < 0.001) (Figure 2B). The gene editing efficiency observed in the two transformed cultures was 64.8% and 86.3%, and the gene editing efficiency observed in the culture transformed with pCas-SSB/pTargetF-*lacZ* was 33.2% higher than that in the culture transformed with pCas-T4L/pTargetF-*lacZ* (*p* < 0.001) (Figure 2C). In each group, 10 white colonies were randomly picked from the plates and were subjected to colony PCR sequencing for identification. The indels in the mutant *lacZ* genes that were generated by pCas-T4L/pTargetF-*lacZ* transformation were characterized by a deletion of one–three bases upstream of the PAM sequence CGG. The deletion sizes in the transformants ranged from 32 to 252 bp, with an average of 104 bp. The deletion junction in five samples had a 1 or 2 bp microhomology region (Figure 2D). The indels in mutant *lacZ* genes that were generated by pCas-SSB/pTargetF-*lacZ* transformation were characterized by a deletion of one–three bases upstream of the PAM sequence CGG. The deletion sizes in the transformants ranged from 45 to 214 bp, with an average of 121 bp. The deletion junction in six samples had a 1–3 bp microhomology region (Figure 2E).

### 3.3. SSB Mediated DSB Repair Independently of RecA and RecBCD

**Figure 3:**
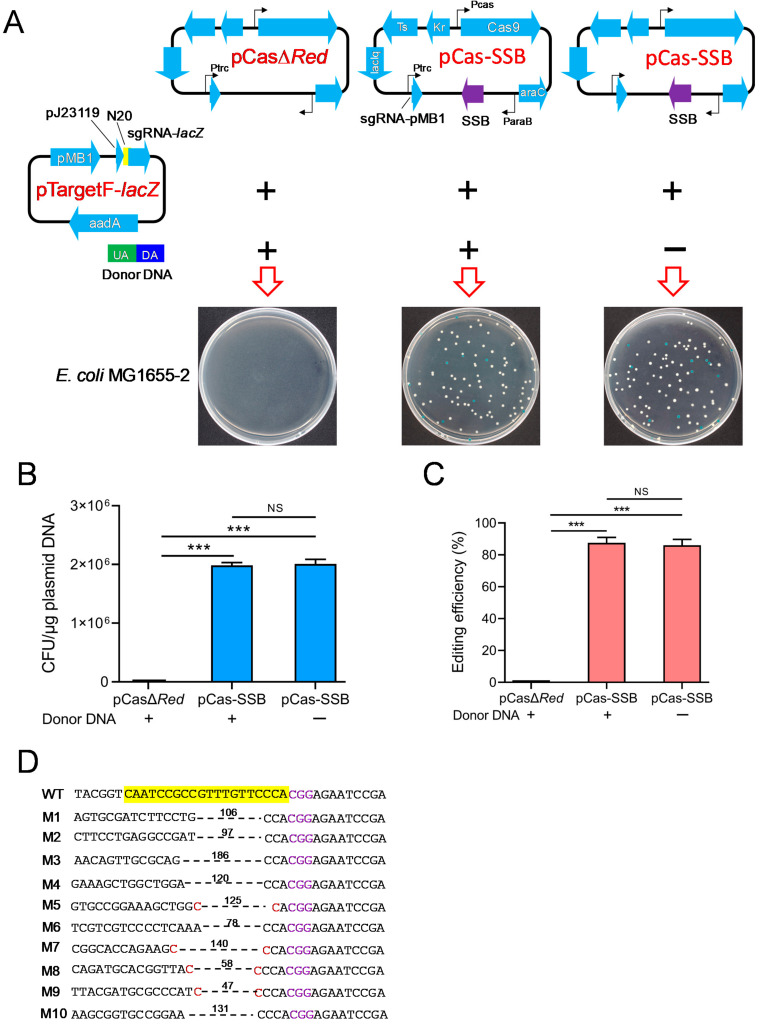
SSB repaired the DSBs and improved gene editing induced by CRISPR-Cas9 independent of RecA and RecBCD. (**A**) Schematic of the CRISPR-Cas9 two-plasmid system transformation into *E. coli* MG1655-2 (Δ*recA*, Δ*recBCD*, Δ*SSB*) to delete the *lacZ* gene with or without donor DNA. (**B**) The transformation rate of the CRISPR-Cas9 plasmids into *E. coli* MG1665-2 with or without donor DNA. (**C**) The editing efficiency of CRISPR-Cas9 to delete the *lacZ* gene in *E. coli* MG1655-2 with or without donor DNA. (**D**) Identification of the 10 randomly selected white colonies of *E. coli* MG1655-2, generated by pCas-SSB and pTargetF-*lacZ* transformation without donor DNA, by colony PCR and DNA sequencing. The sequences highlighted in yellow represent a 20-bp complementary region (N_20_) matching with the *E. coli lacZ* gene. The sequences colored in purple indicate the PAM sequence of Cas9. The sequences colored in red are micro-homology sequences. The dotted lines and associated numbers indicate the deleted sequences and deletion sizes (bp). UA, upstream homologous arm; DA, downstream homologous arm; WT, *E. coli* MG1665; M1-M10, the 10 randomly selected white colonies. The data in (**B**,**C**) represent the mean ± s.d. for *n* = 3 biologically independent samples. ***, *p* < 0.001 by one-way ANOVA followed by Dunnett’s test; ns, no significant differences.

To validate whether SSB mediated the DSB repair independently of RecA and RecBCD in *E. coli*, pCasΔRed/pTargetF-*lacZ* or pCas-SSB/pTargetF-*lacZ* as well as donor DNA as needed were transformed into *E. coli* MG1655-2 (Δ*recA*, Δ*recBCD*, Δ*SSB*). In the culture that was transformed with pCasΔ*Red*/pTargetF-*lacZ* and donor DNA, no colonies grew on the LB plates supplemented with X-gal, IPTG, kanamycin, and spectinomycin. In the culture that was transformed with pCas-SSB/pTargetF-*lacZ* with donor DNA or pCas-SSB/pTargetF-*lacZ* without donor DNA, white and blue colonies grew on the plates (Figure 3A). The transformation rate of both cultures was 2.0 × 10^6^ CFU/μg plasmid DNA, and the gene editing efficiency of the two transformed cultures was 86.0% and 87.6%; that is, no difference was observed between these two cultures (Figure 3B,C). In each transformation group, 10 white colonies were randomly picked from the plates and were subjected to colony PCR sequencing for identification. In all the mutants that were generated by transformation with pCas-SSB/pTargetF-*lacZ* and donor DNA, the *lacZ* gene was correctly knocked out by HR, and this effect was also observed in *E. coli* MG1655 (Figure 1D). The indels in the mutant *lacZ* genes that were generated by transformation with pCas-SSB/pTargetF-*lacZ* without donor DNA were characterized by a deletion of two–three bases upstream of the PAM sequence CGG. The deletion sizes in the mutant transformants ranged from 47 to 186 bp, with an average of 109 bp. The deletion junction in four samples had a 1 bp microhomology region (Figure 3D).

### 3.4. SSB-Mediated HR Promoted the Deletion of the Pseudomonas sp. UW4 wp116 Gene by CRISPR/Cas9

**Figure 4:**
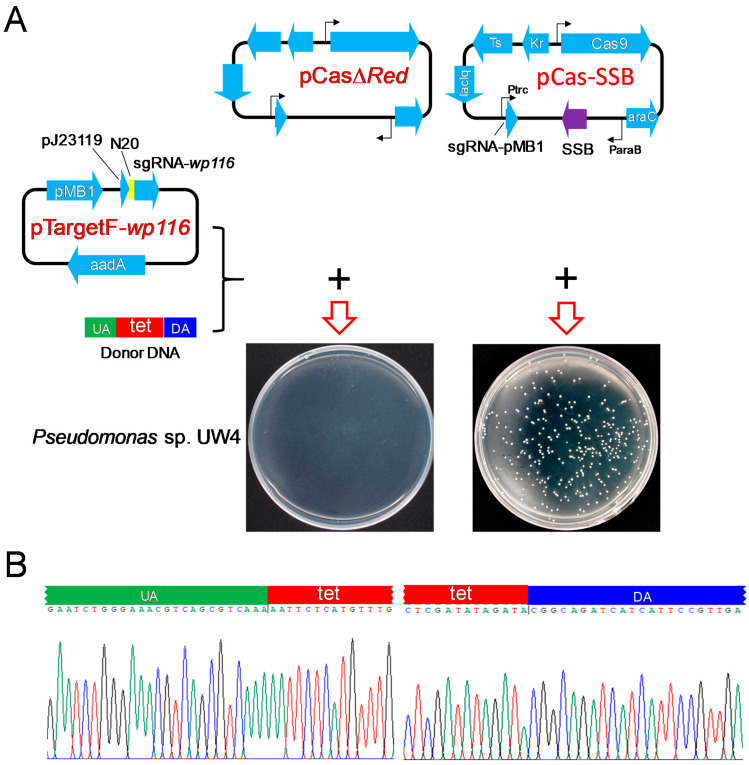
CRISPR-Cas9 editing of the *Pseudomonas* sp. UW4 *wp116* gene assisted by SSB-mediated HR repair of DSBs. (**A**) Illustration of the plasmid expression of Cas9 and sgRNA targeting the *wp116* gene and their transformation into *Pseudomonas sp.* UW4. (**B**) Identification of the 10 randomly selected transformant colonies of *Pseudomonas* sp. UW4 by colony PCR and DNA sequencing. UA, upstream homologous arm, homologous to the upstream region of the *wp116* gene in the UW4 genome; DA, downstream homologous arm, homologous to the downstream region of the *wp116* gene in the UW4 genome; tet, tetracycline resistance gene.

To examine whether SSB effectively mediates HR to promote CRISPR/Cas9 gene editing in bacteria other than *E. coli*, donor DNA harboring a tetracycline-resistance gene cassette and sequences located at both ends of the cassette, complementary sequences to the upstream and downstream regions of the *Pseudomonas* sp. UW4 *wp116* gene, and pCasΔ*Red*/pTargetF-*wp116* or pCas-SSB/pTargetF-*wp116* were transformed into *Pseudomonas* sp. UW4. When *Pseudomonas* sp. UW4 was transformed with donor DNA and pCasΔ*Red*/pTargetF-*wp116*, no colonies grew on the LB plates supplemented with tetracycline, kanamycin, and spectinomycin. After transformation with donor DNA and pCas-SSB/pTargetF-*wp116*, colonies grew on the LB plates (Figure 4A), and the transformation rate was 3.3 × 10^6^ CFU/μg plasmid DNA. Twenty randomly selected colonies were identified by colony PCR sequencing. The *wp116* gene in all the colonies was correctly deleted by HR (Figure 4B), revealing that the gene editing efficiency achieved 100%.

## 4. Discussion

All SSB proteins harbor at least one DNA-binding oligonucleotide/oligosaccharide-binding (OB) fold. The OB fold controls both ssDNA binding and oligomerization. Furthermore, SSBs also play key roles in recruiting SSB/ssDNA-processing enzymes that mediate DNA replication, recombination, and repair [42]. SSB and RecBCD from *E. coli* were demonstrated to be capable of catalyzing dsDNA HR in vitro. Both the HR and NHR efficiencies of *E. coli* expressing inactive *SSB* or *recA* were notably reduced. When *SSB* and *recA* were simultaneously lacking, cells lost their HR and NHR capabilities, revealing that SSB catalyzes HR and NHR with the assistance of nucleases [44]. In this study, we found that SSB-mediated HR and NHEJ efficiently repaired CRISPR/Cas9-induced DSBs, increased the number of viable colonies, and increased the gene editing efficiency in *E. coli* and *Pseudomonas*, even in *recA-*, *recBCD-* and *SSB*-deficient *E. coli*. It was further confirmed that SSB catalyzed HR and NHR with the aid of a nuclease.

The λ-Red recombinase system is commonly used to repair CRISPR/Cas9-induced DSBs and improve CRISPR/Cas9-assisted genome editing efficiency, and homologous donor ssDNA or dsDNA is required [26,27,33]. Compared with the λ-Red recombinase system, in this study, replacing the λ-Red recombinase system with SSB and providing donor dsDNA did not change the transformation rate but increased the gene editing efficiency by 21.4%. Surprisingly, the gene editing efficiency of SSB-mediated HR increased to 100% in *Pseudomonas*. SSB is composed of only 178 amino acids, and it is much smaller than λ-Red recombinases.

The efficiency of CRISPR/Cas9 genome editing via phage T4 DNA ligase-mediated repair of DSBs was markedly higher than that achieved via the mycobacteria-derived NHEJ pathway [28,32]. Compared to T4 DNA ligase-mediated repair of DSBs by NHEJ, the transformation rate and genome editing efficiency achieved via SSB-mediated repair of DSBs by NHEJ were 52.6% and 33.2% higher, respectively; however, the length of deleted chromosomal DNA and the deletion junction manner did not differ between these groups. Inducing the expression of Cas9 and assisting native A-EJ DNA repair could efficiently delete chromosomes in *E. coli* [29]; however, the process is complicated and time-consuming, and it has a high rate of off-target effects. These results suggested that the SSB-mediated HR and NHEJ mechanism for DSB repair that is described here provides an effective approach to improve CRISPR/Cas9 genome editing efficiency in *E. coli* and *Pseudomonas*.

It was reported that the Redβ protein of the λ Red recombination system alone can repair DSBs with ssDNA donors and that it can facilitate CRISPR/Cas9 genome editing [30]. Nevertheless, ssDNA donors must be artificially synthesized and are easily enzymatically digested. The Redβ protein contains a small OB fold [50], similar to SSB, which enables ssDNA binding, protects ssDNA from damage, reduces secondary structure formation in single-stranded DNA, and stimulates the formation of joint molecules by the RecA protein from linear duplex DNA and homologous circular single strands; however, unlike SSB, the Redβ protein cannot promote heteroduplex joint formation (dsDNA recombination) [51]. Furthermore, the Redβ protein can initiate single-strand annealing homologous DNA recombination and strand invasion [52,53]. Therefore, there is some difference between the SSB and Redβ proteins in terms of the mechanism by which they repair the DSBs that are induced by CRISPR/Cas9.

With diverse metabolic pathways and efficient metabolic rates, bacteria can synthesize novel and economically important products, such as enzymes, organic acids, vitamins, antibiotics, antibodies, hormones, carotenoids, steroids, alkaloids, alcoholic beverages, interferons, and vaccines [54]. CRISPR/Cas technology has been proven to be a very robust and effective technique for editing bacterial genomes and optimizing bacterial metabolic pathways. However, the first challenge of this approach is to ensure that the engineered cells survive CRISPR/Cas-induced DSBs [55]. The use of SSB for efficient repair of CRISPR/Cas-induced DSBs provides a better option for bacterial pathway engineering.

In summary, *E. coli* SSB-mediated HR and NHEJ efficiently repaired CRISPR/Cas9-induced DSBs to increase the number of viable colonies and the gene editing efficiency in *E. coli* and *Pseudomonas*. These processes occurred independently of RecA and RecBCD. The efficiency of CRISPR/Cas9 genome editing assisted by SSB was higher than that of the λ-Red recombinase system via HR and T4 DNA ligase via NHEJ. These results showed that SSB-assisted CRISPR/Cas9 genome editing is an alternative approach to facilitate the editing of bacterial genomes. To the best of our knowledge, this is the first report that *E. coli* SSB can repair the DSBs caused by CRISPR/Cas9 and effectively improve CRISPR/Cas9 genome editing in *Escherichia coli* and *Pseudomonas*.

## Figures and Tables

**Table 1:** Strains and plasmids used in this study ^a^.

Strain or Plasmid	Description	Source or Reference
*E. coli*		
DH5α	F^−^ *endA1 glnV44 thi-1 recA1 relA1 gyrA96 deoR nupG* ϕ80d*lacZ*ΔM15 Δ(*lacZYA-argF*)*U169 hsdR17* (r_K_^−^ m_K_^+^) λ^−^	TaKaRa
MG1655	K-12; F^−^, λ^−^, *ilvG*^−^, *rfb-50, rph-1*	[45]
MG1655-1	MG1655 Δ*lacZ*	This study
MG1655-2	MG1655 Δ*recA* Δ*recBCD* Δ*SSB*	This study
*Pseudomonas* sp.		
UW4	Wild type	[46]
UW4 Δ*acdS*	Δ*acdS*	[47]
UW4 Δ*wp116*	Δ*wp116*	This study
Plasmids		
pCas	*repA101*(Ts) *kan P_cas_-cas9 P_araB_-Red lacI*^q^ *P_trc_*-sgRNA-*pMB1*	[33]
pCasΔ*Red*	*repA101*(Ts) *kan P_cas_-cas9 P_araB_ lacI*^q^ *P_trc_*-sgRNA-*pMB1*	This study
pCas-SSB	*repA101*(Ts) *kan P_cas_-cas9 P_araB_-SSB lacI*^q^ *P_trc_*-sgRNA-*pMB1*	This study
pCas-T4L	*repA101*(Ts) *kan P_cas_-cas9 P_araB_-T4L lacI*^q^ *P_trc_*-sgRNA-*pMB1*	This study
pTargetF	*pMB1 aadA* sgRNA	[33]
pTargetF-*lacZ*	*pMB1 aadA* sgRNA-*lacZ*	This study
pTargetF-*wp116*	*pMB1 aadA* sgRNA-*wp116*	This study

^a^ *kan*, kanamycin resistance gene; *aadA*, spectinomycin resistance gene; *P_cas_-cas9*, the *cas9* gene with its native promoter; *P_araB_-Red*, the *Red* recombination genes with an arabinose-inducible promoter; *P_trc_*-sgRNA-*pMB1*, sgRNA with an N_20_ sequence for targeting the *pMB1* region with a *trc* promoter; sgRNA-*lacZ*, sgRNA with an N_20_ sequence for targeting the *lacZ* locus; sgRNA-*wp116*, sgRNA with an N_20_ sequence for targeting the *wp116* locus; *wp116*, the gene encoding the chemotactic receptor for 1-aminocyclopropane-1-carboxylic acid (ACC); *SSB*, the gene encoding the single-stranded DNA-binding protein of *E. coli*; T4L, T4 DNA ligase; acdS, 1-amino-cyclopropane-1-carboxylate deaminase.

**Table 2:** The primers used in this study ^a^.

Primer	Sequence (5′-3′)
pC01	CGCATCCTCACGATAATATCCGGGTAGGCGCAATCACTTT
pC02	GATATTATCGTGAGGATGCGTTTTTATAACCTCCTTAGAG
pC03	TCGAGCTCTAAGGAGGTTATAAAAAATGGCCAGCAGAGGCGTAAACAAGGT
pC04	ACCCGGATATTATCGTGAGGATGCGTCAGAACGGAATGTCATCATCAAAGT
pC05	CGCATCCTCACGATAATATCCGGGT
pC06	TTTTTATAACCTCCTCCTTAGAGCTCG
pC07	TCGAGCTCTAAGGAGGTTATAAAAAATGATTCTTAAAATTCTGAACGAAAT
pC08	ACCCGGATATTATCGTGAGGATGCGTCATAGACCAGTTACCTCATGAAAAT
pC09	CAATCCGCCGTTTGTTCCCACGGGTTTTAGAGCTAGAAATAGCAAGTTAAAATAA
pC10	TGGGAACAAACGGCGGATTGACTAGTATTATACCTAGGACTGAGCTAGCTG
pC11	CCGCGGCCTGATCGAACAACGTTTTAGAGCTAGAAATAGCAAGTTAAAATAA
pC12	GTTGTTCGATCAGGCCGCGGACTAGTATTATACCTAGGACTGAGCTAGCTG
pC13	CGGTAGTGGGATACG
pC14	CCTGCCCGGTTATTAAGCTGTTTCCTGTGT
pC15	ACACAGGAAACAGCTTAATAACCGGGCAGG
pC16	AAAAGCCTAGATAAA
pC17	AGCTATTCGCCCATACATCG
pC18	CCTGCCCGGTTATTAAGCTGTTTCCTGTGT
pC19	GGAAACGTCAGCGTCAAAAATTCTCATGTTTGACAG
pC20	CGGAATGATGATCTGCCGTATCTATATCGAGATGCG
pC21	CGCATCTCGATATAGATACGGCAGATCATCATTCCG
pC22	CTTGGGTCATGCTCTGCATGG
pC23	CGGTAGTGGGATACGACGAT
pC24	CGGTTGGAATAATAGCGAGA
pC25	CCCAGGCTTTACACTTTATGC
pC26	CAGATGAAACGCCGAGTT
pC27	AGCTATTCGCCCAT
pC28	CTTGGGTCATGCTCT

^a^ The sequences in blue are N_20_ sequences.

## Data Availability

Not applicable.
